# Hydrogen adsorption on doped MoS_2_ nanostructures

**DOI:** 10.1038/s41598-017-15622-z

**Published:** 2017-11-10

**Authors:** Mikko Hakala, Rasmus Kronberg, Kari Laasonen

**Affiliations:** Department of Chemistry and Materials Science, School of Chemical Engineering, Aalto University, P.O.Box 16100, FI-00076 Aalto, Finland

## Abstract

Electrochemical devices for efficient production of hydrogen as energy carrier rely still largely on rare platinum group metal catalysts. Chemically and structurally modified metal dichalcogenide MoS_2_ is a promising substitute for these critical raw materials at the cathode side where the hydrogen evolution reaction takes place. For precise understanding of structure and hydrogen adsorption characteristics in chemically modified MoS_2_ nanostructures, we perform comprehensive density functional theory calculations on transition metal (Fe, Co, Ni, Cu) doping at the experimentally relevant MoS_2_ surfaces at substitutional Mo-sites. Clear benefits of doping the basal plane are found, whereas at the Mo- and S-edges complex modifications at the whole edge are observed. New insight into doping-enhanced activity is obtained and guidance is given for further experiments. We study a machine learning model to facilitate the screening of suitable structures and find a promising level of prediction accuracy with minimal structural input.

## Introduction

The concept of hydrogen economy comprises the idea to produce, store, distribute and use hydrogen as renewable fuel^1^. In this technology hydrogen can be cleanly produced by electrolytic splitting of water to hydrogen and oxygen if the process is powered by renewable energy sources^1,2^. However, the water-splitting process relies currently on catalysts comprised of platinum group metals (PGMs), which are considered as critical raw materials in terms of supply^3^. The metal dichalcogenide MoS_2_ has been suggested experimentally and theoretically as a promising candidate to replace the PGMs for the hydrogen evolution reaction (HER) at the cathode side^2,4^. The recent steps in the development (see, for example^5–10^,) have been to modify it structurally, e.g., by synthesizing various types of nanostructures and chemically, e.g., by doping, which are both procedures to maximize the area of the active surface/edge configurations and sites to obtain optimal HER performance. For guiding and supporting the experimental search of replacement materials, detailed theoretical information on the chemically and structurally modified nanostructures is essential. The Gibbs free energy of adsorption Δ*G*_H_ for the reaction intermediate, i.e., hydrogen at the electrode surface, has been a widely used descriptor for predicting catalytic performance based on experimental correlations and mathematical models (Refs^11,12^ and references therein). It has been used for various transition metal dichalcogenides and doped MoS_2_ previously^7,13,14^.

Synthesized MoS_2_ nanostructures have differently S-covered edges at various proportions, lengths and distributions depending on the preparation method^6,15,16^. The structures can also contain less regular parts such as defects and terrasses. Importantly, each geometrically and chemically different part may correspond to specific HER efficiency. The undoped, pristine basal plane of 2H-MoS_2_ is understood to be inactive^4,13,17^. Several theoretical studies have been devoted to the pristine Mo and S-edges of MoS_2_ in terms of Δ*G*_H_. Especially the Mo-edges are considered as active: the 100% S-covered Mo-edge of nanoclusters^6^ and the 50% S-covered Mo-edge in industrial-style catalysts^18^. Regarding modification with doping, Kibsgaard *et al*.^6^ studied Fe, Co, Ni and Cu and obtained truncated triangle-shaped nanoclusters, finding Ni the best and Co the second best for promoting HER activity. In their clusters doping itself changes the morphology of the cluster (the relative linear lengths of the Mo- and S-edges) and thereby the activity. Šarić *et al*.^19^ studied by density functional theory (DFT) calculations the corresponding Co-doped nanoclusters. Escalera-Lopez *et al*.^20^ reported Ni-MoS_2_ hybrid nanoclusters which showed a roughly 3-fold increase in exchange current density compared with undoped nanoclusters. They associated the findings to Ni-doped Mo-edge and S-edge sites. Deng *et al*.^14^ performed experiments on the doped basal plane of MoS_2_ and found the trend for HER activity as Pt (highest) > Co > Ni as dopants. They found a similar trend in their DFT calculations for various dopants. Li *et al*.^21^ studied single Pt atomic structure and dynamics in monolayer MoS_2_ experimentally and by DFT calculations. Dai *et al*.^22^ reported enhanced electrocatalytic properties for Co-doped MoS_2_ nanosheets and attributed the finding to doping at the Mo and S edges. Wang *et al*.^7^ reported DFT calculations for Δ*G*_H_ of Mo- and S-edges for pristine and TM-doped (Fe, Co, Ni, Cu) MoS_2_. They also synthesized and characterized doped vertically aligned nanofilms which expose alternatingly infinite Mo- and S-edges. Their results for the doped S-edge suggested enhanced catalytic activity as close to optimal (Δ*G*_H_ = 0 eV) values of hydrogen adsorption were found compared to the undoped edge. Finally, doped (Fe, Co, Ni) amorphous MoS_2_ was studied by Morales *et al*.^5^.

**Figure 1:**
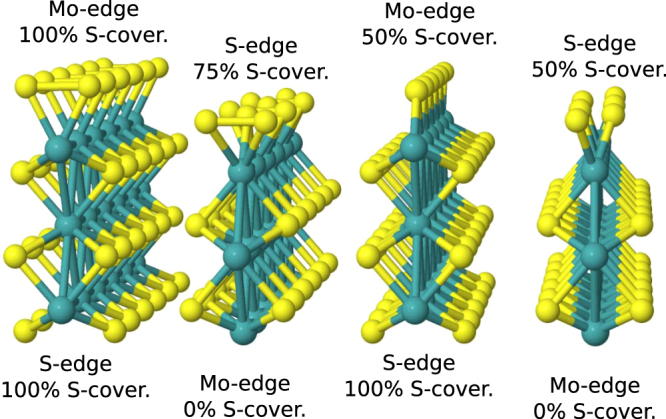
MoS_2_ edge structures. The sulfur coverage of the edges is indicated.

In this work we provide a systematic study of the hydrogen adsorption structures and energetics for Fe, Co, Ni and Cu-doped 2 H basal plane and Mo- and S-edges at low H coverage conditions to clarify the precise effect of chemical modification of MoS_2_. For comparison with earlier work, additional calculations are performed for Pd and Pt dopants and for higher H coverages. All the systems are calculated using the same level of description and without structural constraints, which provides a unique set of data. For Mo we study the 0%, 50% and 100% sulfidized edges and for S the 50%, 75% and 100% ones. The edge structures are illustrated in Fig. 1, denoted hereafter Mo-*X* or S-*X*, where *X* indicates the degree of sulfur coverage in percents. We calculate relative substitutional energies (RSEs) to assess the affinities of doping at different edges and analyze the local structural changes. By using the calculated Δ*G*_H_ as a descriptor and comparing the results with experiments, we discuss the suitability of doping in the various cases for improving the HER activity. Since the detailed structure-property relationship, directly or indirectly via Δ*G*_H_, i.e., [atomic and electronic structure of the surface] → Δ*G*_H_ → [*i*_0_, exchange current density], is far from trivial, our results offer new interpretations, suggestions and trends for experimental synthesis and for further theoretical work. For facilitating fast prediction of Δ*G*_H_ values (i.e., bypassing the DFT step), we illustrate a supervised machine learning (ML) model, which also informs about the importance of the structural features that determine the strength of H adsorption. Low H coverage is obtained in supercell calculations by adsorbing single H atoms on target areas. Studying this regime for the edge structures is consistent with the finding by Wang *et al*.^7^ for the Tafel slopes in their doped nanofilm experiments, which indicated that the rate-limiting HER step is the Volmer reaction, which corresponds to low H surface coverage. We will monitor Δ*G*_H_ not only in reference to the optimal condition (Δ*G*_H_ = 0 eV), but for description and classification consider also Δ*G*_H_’s that are found within a range of values (such as −0.5 eV < Δ*G*_H_ < 0.5 eV). In this work we perform the calculations in electrically neutral supercells, but to assess the possible effects of non-neutral charge states, we also analyze explicitly two cases for an illustrative example: the doped basal plane and the undoped and Fe-doped S-100 edge in charge states +1, −1 and −2 of the supercell.

The rest of the paper is organized as follows. We first present the structural and hydrogen adsorption results for the doped 2 H basal plane and then for the doped Mo- and S-edges. Next, we demonstrate the classification and regression models for predicting Δ*G*_H_ values and find the importances of the input variables. Before concluding, we discuss the more general implications, ideas and suggestions to experimental synthesis of MoS_2_ nanostructures.

## Results

### 2H basal plane

#### Effect of doping on structure

**Figure 2:**
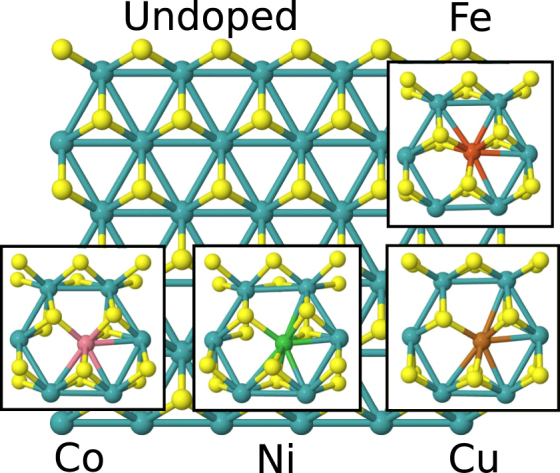
Basal plane of 2H-MoS_2_ and its local structural deformations (insets) when Fe, Co, Ni and Cu are doped at substitutional Mo sites. View from the top.

The effect of substitutional doping on the local geometry of the basal plane is illustrated in Fig. 2. The doping level is 2.8% in the plane (that is, 1 of 36 Mo atoms in the topmost surface layer is replaced by the dopant in the supercell). For Fe the local geometry has a slightly reduced symmetry compared to the pristine structure, whereas a clearer symmetry-breaking effect occurs for Co, Ni and Cu, leading to a 5-fold coordinated structure. A similar symmetry breaking is found for Pd and Pt. Numerical values for the nearest-neighbor distances are reported in Supplementary Information. Our results for the local geometry are to some extent different from those reported previously by Deng *et al*.^14^. Instead of our 5-fold coordination for Co, Ni, Cu, Pd and Pt, they found a 4-fold coordinated structure. These differences are discussed below further in the case of Δ*G*_H_ values. Finally, we note that when H adsorbs on the sulfur next to the Fe, Co, Ni and Cu dopant, no further essential symmetry breaking occurs, the only exception being that also Fe becomes 5-fold coordinated. It is possible that there are non-substibutional and other available sites for the dopant atoms, but at least the calculations by Deng *et al*.^14^ suggest that they may have much higher formation energies than the substitutional one.

To study the charge state effects on the doped structures we carry out a standard formation energy analysis (see Methods). The analysis provides the relative stabilities of the doped systems at different charge states as a function of the electron chemical potential *μ*_*e*_ (*μ*_*e*_ measured from the valence band maximum). For Fe, Co and Ni the the results show that the neutral charge state is the most stable roughly in the range 0 eV $\le {\mu }_{e}\mathop{ < }\limits_{ \tilde {}}0.6$ eV above which the negative charge states becomes the most likely. For Cu, the negative charge state becomes more likely above ${\mu }_{e}\sim 0.2$ eV. These findings do not hint at major changes in the oxidation number of the dopants that substitute Mo. Moreover, since ${\mu }_{e}\mathrm{ > 0.6}$ eV corresponds in principle already to a high cathodic overpotential, systems in that range of *μ*_*e*_ are not any more in the optimal target area for good HER catalysts.

The modification of the electronic structure and the subsequent effects on geometry due to TM doping can be understood considering the *d* electrons in the local molecular symmetry. In pristine 2H-MoS_2_ Mo is in the oxidation state IV, has two 4*d* electrons and is coordinated to six nearest-neighbor sulfur atoms. According to Ref.^23^ the system has trigonal prismatic symmetry with point group ${D}_{3h}$. The two 4*d* electrons of Mo(IV) occupy a bonding *d* orbital with ${a}_{1}$ symmetry on top of the valence band, and the four unoccupied (non-bonding *e* and antibonding $e^{\prime}$) *d* states are higher in energy^23^. By doping TM atoms substitutionally at the Mo site brings, as a first approximation, excess *d* electrons to the local structure. In addition, TM–S bond lengths contract compared with the Mo–S lengths due to a smaller atomic size of the TM atom. The excess *d* electrons start to occupy progressively the previously empty $e$ and $e^{\prime}$ states. As discussed above, for Co, Ni and Cu (5, 6 and 7 *d* electrons, respectively) the systems breaks to a lower 5-fold symmetry (see Fig. 1), and this can be directly correlated with the antibonding $e^{\prime}$ state becoming occupied. In short, progressive occupancy of the local antibonding *d* states triggers the symmetry-lowering transition.

#### Effect of doping on H adsorption

A simple way to describe doping effects is that modifying the pristine Mo-S structure changes the binding of S at the surface. This consequently modifies the hydrogen adsorption free energy Δ*G*_H_ and provides a route to tune the HER activity. In the case of TM dopants (Fe, Co, Ni) at substitutional Mo sites, the specific occupancy of their local bonding and antibonding 3*d* states has been used as the explanation for the weaker binding between the dopant and sulfur^24^.

Our results show that for the basal plane, doping with Fe, Co, Ni and Cu (as well as with Pd and Pt) is clearly beneficial for bringing Δ*G*_H_ values close to optimal adsorption condition (${\rm{\Delta }}{G}_{{\rm{H}}}\approx 0$ eV) at the sulfur site that is the nearest-neighbor to the dopant. For Ni, three local minima at the sulfur next to the dopant site were found during the search. This suggests that the local potential energy landscape in general is rich in detail but the adsorption energies are still rather close to each other. Farther away from the dopants larger Δ*G*_H_ values are found. Δ*G*_H_ values in the (−1) charge state are found to be similar within about ±0.1 eV to those of the neutral charge state (the largest difference 0.2 eV in the case of Cu).

The results (Table 1) demonstrate that the major improvement in Δ*G*_H_ values remains local close to the substitutional dopant atom. A similar finding was reported by Deng *et al*.^14^ for Pt. Naturally, the higher the substitutional dopant density at the outermost surface layer and without causing other structural modifications, the better the overall HER activity. Our Δ*G*_H_ values for the nearest-neighbor adsorption are slightly different from those reported by Deng *et al*.^14^. For Fe, Pd and Pt, their Δ*G*_H_ values are ~ 0.2–0.4 eV higher compared to ours and for the rest of the cases (undoped, Co, Ni and Cu) the values are similar. These differences must be attributed to different computational choices (most probably concerning the size and structure of the supercell), which leads to a different detailed relaxation geometry. Our result for the pristine basal plane ${\rm{\Delta }}{G}_{{\rm{H}}}=1.88$ eV is also close to the previously reported differential hydrogen free energy of adsorption ${\rm{\Delta }}{G}_{{\rm{H}}}^{diff}$ of 1.92 eV at low H coverage^25^.

One can also search from our results structure-based hints that could predict optimal adsorption energies. From Table 1 for Fe…Ni it can be deduced that there is a negative correlation between the number of the valence shell electrons and Δ*G*_H_ values. We also found some dependence of Δ*G*_H_ on the Löwdin charge of the sulfur site before H adsorption. However, this latter dependency would be difficult to utilize for fast screening since it itself requires a DFT calculation. Predictive and powerful approaches can be probably best built using machine learning methods by encoding the geometries into multidimensional input attributes^26^. For initial insight, in this work we employ a straightforward classification and regression analysis for Δ*G*_H_ prediction including as attributes the type of the surface, coordination number, dopant, and the dopant-hydrogen distance.

**Table 1:** Hydrogen adsorption free energy Δ*G*_H_ (eV) on the doped MoS_2_ 2H basal plane. The adsorption site is the nearest neighbor S of the dopant, unless otherwise indicated. *denotes other local minima at the nearest neighbor S in the case of Ni. “*n* th nn” refers to the *n* th nearest neighbor. The values by Deng *et al*.^14^ are given (approximate values estimated from their figures unless given in supporting material). Δ*G*_H_ values at charge state (−1) are also given for comparison.

dopant		Δ*G*_H_	
		this work	Deng *et al*.
pristine		1.88	1.83
Fe		0.22	0.6
Fe (−1)		0.37	—
Co		−0.09	−0.07
Co (−1)		−0.02	—
Ni		−0.33	−0.28
Ni*		−0.13, −0.01	—
Ni (−1)		−0.34	—
Cu		−0.10	−0.2
Cu (−1)		−0.30	—
Pd		−0.22	−0.05
Pt		−0.24	−0.00
Co	3rd nn	1.74	—
Ni	2nd nn	1.18, 1.34	—
Ni	4th nn	1.46	—
Pt	2nd nn	1.63	1.35

### Mo- and S-edges

#### Effect of doping on structure

**Figure 3:**
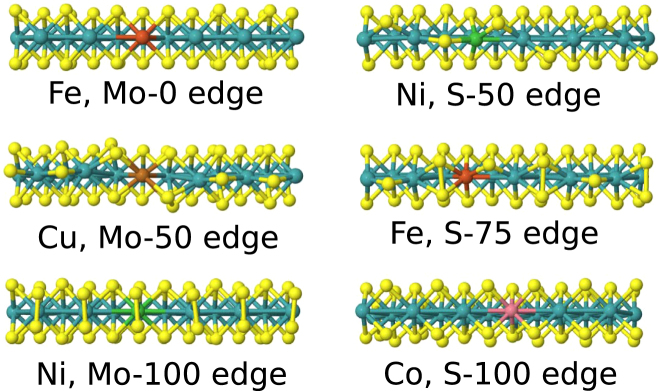
Examples of relaxed structures for substitutionally doped MoS_2_ edges (view from the top).

The studied doping level for the present edge structures is 16.7% (one Mo atom of the six along the edge row substituted). This is of the order of experimental values in the work of Wang *et al*.^7^, who found by XPS that in Co-doping the Co/Mo ratio is ~ 0.29 at the outermost row of the edge structures in their vertically aligned samples, which corresponds to the doping level of ~ 25% of the edge. They found that the ratio decreases continuously deeper from the edge. For small nanoclusters, Kibsgaard *et al*.^6^ concluded a 100% substitutional doping level at the S-edge. Our results are for supercells with stacked MoS_2_ layers which expose alternatingly the different edges (the system is periodic along the edges and in the direction of the plane normals, see example in Supplementary Information). The edges of these systems should thus correspond closely to the experimental samples discussed by Wang *et al*.^7^. As a structural detail, we note that the Δ*G*_H_ values for H adsorption on the edges of *isolated* infinite MoS_2_ sheets (periodic in one dimension) differ from the ones presented here; a few test calculations revealed differences in Δ*G*_H_ of the order of ±0.3 eV. The effect of doping on the local symmetry and dopant energetics are discussed first before addressing hydrogen adsorption. The changes in the local symmetry due to substitutional doping are summarized in Table 2 (discussed in Supplementary Information in more detail). Examples of the relaxed edges are given in Fig. 3.

**Table 2:** Summary of changes in local symmetry due to substitutional doping at the MoS_2_ edges. ‘no change in symmetry’ denotes no essential changes except possibly in bond lengths. Fe (*q*) denotes supercell calculations in non-neutral charge states.

	no change in symmetry	slightly reduced symmetry	stronger deformation
Mo-0	Fe, Co, Ni, Cu	—	—
Mo-50	Fe, Co, Ni	—	Cu
Mo-100	Fe, Co, Ni	—	Cu
S-50	Fe, Co	Ni, Cu	—
S-75	Co, Ni	Fe	Cu
S-100	Fe, Fe (*q*), Co, Ni	—	Cu

The charging effects on the edge structures are studied for the undoped and Fe-doped S−100 structures as examples in the charges states +1, −1 and −2. The formation energy analysis shows that at the electron chemical potential in the range 0 eV $\le {\mu }_{e}\mathop{ < }\limits_{ \tilde {}}0.6$ eV also the negative supercells (charge states −1, −2) are energetically relevant for both the cases. For the undoped edge also the +1 state is energetically relevant. This indicates that the edge systems themselves without and with dopants can trap charge, although no major changes can be observed in the atomic structures of the supercells. For the purposes of this work, the interesting question is to what extent charge states affect the Δ*G*_H_ values for H adsorption. This will be discussed in the corresponding section below.

#### Relative probability of doping at the edges

In the case of nanocluster model catalysts by Kibsgaard *et al*. (diameter ~65 Å at maximum)^6^, there is strong evidence that the dopants have distinct tendencies to become incorporated into the different edges. Theoretical calculations by Schweiger *et al*. have elucidated the possible dopant energetics and morphologies in various sulfiding environments^27^. Similar kind of energetics information for the probability of doping is now extracted from the present total energy calculations. If a given MoS_2_ nanostructure contains edges, the comparisons suggest onto which edge the dopant has the largest affinity. The results for relative substitutional energies (RSEs) are collected in Table 3 . The reference level corresponds to substituting the Mo atom by a dopant atom on the MoS_2_ basal plane. This reference is given by the energy difference ${E}_{{\rm{r}}{\rm{e}}{\rm{f}}}={E}_{{\rm{T}}{\rm{M}}-{\rm{d}}{\rm{o}}{\rm{p}}{\rm{e}}{\rm{d}}{\rm{b}}{\rm{a}}{\rm{s}}{\rm{a}}{\rm{l}}{\rm{p}}{\rm{l}}{\rm{a}}{\rm{n}}{\rm{e}}}-{E}_{{\rm{b}}{\rm{a}}{\rm{s}}{\rm{a}}{\rm{l}}{\rm{p}}{\rm{l}}{\rm{a}}{\rm{n}}{\rm{e}}}$. For each dopant, the lower the RSE for a particular edge, the more probably that edge is doped over the others. We note that in order to compare absolute stabilities of the dopants between each other for a given edge, one should extend the analysis to estimate appropriate bulk references. The current results are thus relevant only for comparing different edges for a given dopant.

**Table 3:** Relative substitutional energies (eV) for TM atoms doped at the edges (dopant concentration 1/6, i.e. 16.7%). The reference is the substitutional energy of doping the basal plane (see text).

	basal plane	Mo-0	Mo-50	Mo-100	S-50	S-75	S-100
Fe	0	−0.38	−1.22	−2.38	−2.80	−3.53	−1.82
Co	0	−4.59	−2.45	−2.88	−4.24	−4.38	−2.15
Ni	0	−5.33	−2.50	−2.51	−4.60	−5.62	−1.88
Cu	0	−5.97	−3.64	−3.42	−5.59	−5.52	−2.61

The fact that the values in the table are negative confirms that from a thermodynamic perspective doping at the edges is systematically easier than doping at the basal plane. In general, our quantitative results are complex and there is a non-trivial dependence on the edge and on the dopant. In the experimental studies of nanoclusters by Kibsgaard *et al*.^6^, the presence of dopants decreases the surface free energy of the S-edge. As a consequence, hexagonally shaped truncated triangles are obtained, instead of plain nanocluster triangles with Mo-100 edges only. These hexagonally shaped structures contain both doped S-edges (S-50 edge in the case of Co and Ni) and undoped Mo-100 edges. According to Kibsgaard *et al*. there is a negative correlation between the relative length of the doped S-edge and the number of valence electrons of the dopant (Fe, Co, Ni and Cu). Although our results in Table 3 are for infinite layered systems and a lower doping level, they also show that doping takes place more likely at the S-50 edge compared with the Mo-100 edge (since RSEs for S-50 are systematically lower than those for Mo-100). Our values also support the assignment proposed in Ref.^6^ that the Fe-doped S-edge is the S-50 edge and not the S-100 edge. If S-100 edges were present, Fe doping would rather take place at the Mo-100 edge (RSE for Mo-100 is lower than that for S-100).

The relevance of Table 3 is general and can be used as a guideline to analyze qualitatively and quantitatively other situations as well, especially the cases when the sulfiding atmosphere is changed^16^. In the following we compare our values to the theoretical energetic calculations by Schweiger *et al*.^27^ for 100% Co- and Ni-doped nanoclusters (triangle-shaped planar systems). The comparison is again semi-quantitative, since our calculations are for extended layered systems and lower doping level. They predicted results for high (${\mu }_{S}\ge$ −0.25 eV), intermediate (−1.1 eV $\ge {\mu }_{S}\ge$ −0.25 eV) and low (${\mu }_{S}\le$ −1.1 eV) chemical potential *μ*_*s*_ of sulfur, corresponding to highly sulfiding, traditional sulfiding and highly reductive environments, respectively. However, the regime of low chemical potential of sulfur is less interesting, since they found a complete destabilization of the nanocluster, which led to the dopants’ segregation into separate phases and suggested this regime to be avoided.

First, for high chemical potential of sulfur Schweiger *et al*.^27^ predicted that both the Mo- and S-edges can be doped. According to them in this case (i) Co is covered 100% by sulfur with 6-fold coordination on both Mo- and S-edges (in other words, the system contains Mo-100 and S-100 edges), and (ii) Ni has 5-fold coordination on the Mo edge and 4-fold coordination on the S edge. Their prediction (i) is consistent with the our RSEs of Table 3: the value for Mo-100 (−2.88 eV) is close to S-100 (−2.15 eV), but far from that of S-50 or S-75 (both close to about −4.3 eV). The closeness of the values suggests that both the Mo-100 and S-100 edges are likely to be simultaneously doped, but for example in the case of the Mo-100 and S-50 edges, the S-50 edge would be preferably doped. Prediction (ii) corresponds to a situation slightly different from our case. In our case Ni has six-fold coordination both at the Mo-50 and Mo-100 edges and the doping is preferred at the S-50 edge.

Second, for intermediate chemical potential of sulfur Schweiger *et al*. considered that pristine MoS_2_ particles have 50% sulfur coverage on both the edges (in other words, the system contains Mo-50 and S-50 edges). For Co doping, they found the nanoparticles to exhibit predominantly the Co-doped S-50 edge, which is consistent with our RSE for S-50 being smaller than that for Mo-50. For Ni doping, they reported the nanocluster to expose the Ni-doped Mo-0 edge and a small fraction of S-50. This prediction is likewise in agreement with our values for Mo-0 having a lower RSE (−5.33 eV) compared with that of S-50 (−4.60 eV).

In general, the results bring new insight into the fundamental differences of how transition metals behave as dopants in MoS_2_.

#### Effect of doping on H adsorption

**Figure 4:**
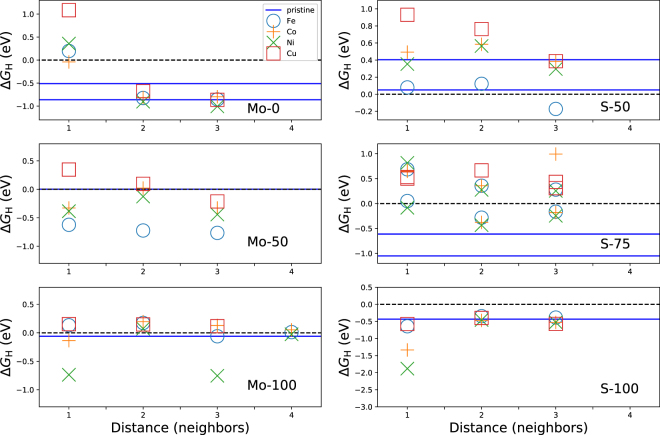
Hydrogen adsorption free energy Δ*G*_H_ on MoS_2_ edges as a function of distance of hydrogen to the dopant atom. The distance is given as the *n*th nearest-neighbor sulfur position relative to the site of the dopant atom (see text). Solid lines(s): adsorption free energy on the pristine edge (for pristine Mo-0, S-50 and S-75 two local sites are identified). Note the different Δ*G*_H_ scales in the panels.

**Figure 5:**
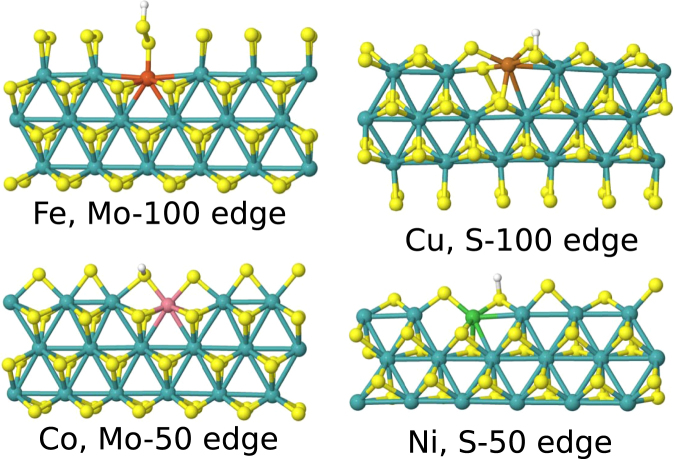
Examples of relaxed structures for hydrogen adsorption on doped MoS_2_ edges (view from the side).

The Gibbs free energy Δ*G*_H_ of adsorbed single hydrogen on the six edges is presented in Fig. 4, in which Δ*G*_H_ is given as a function of the distance from the dopant site (the numerical values are reported in Supplementary Information). Since H adsorbs in most of the cases on top of sulfur, the distance is indexed as the *n* th nearest-neighbor sulfur position relative the dopant atom (the only exception is Mo-0, for which index = 1 corresponds to adsorption on top of the bare metal atom). The values for the pristine edges are marked as horizontal lines (either one or two values are given depending on if more than one minimum is found). Examples of relaxed structures with adsorbed hydrogen are shown in Fig. 5. Some comparison values for Δ*G*_H_ are available from Wang *et al*.^7^ for Mo-50 and S-100, but too direct one-to-one comparisons should be avoided, since they consider differential Δ*G*_H_ values, report results for 100% TM doping (compared to our 16.7%) and have a slightly different structural model and method to calculate the electronic structure.

At the Mo-edges, Mo-0 can be driven toward optimal adsorption conditions with Fe, Co and Ni doping. This could be a relevant route especially in the case of Ni, which is predicted to be stabilized at the Mo-0 edge of triangular nanoclusters at intermediate chemical potential of sulfur^27^. Pristine Mo-50 and Mo-100 exhibit already as such ${\rm{\Delta }}{G}_{{\rm{H}}}\approx 0$ eV values for H adsorption. For Mo-50 we find that doping with any of the four dopants (Fe-Cu) worsens the adsorption energies, which is qualitatively the same conclusions as made by Wang *et al*. For Mo-100 we find a neutral overall effect from Fe, Co and Cu, but from Ni worse Δ*G*_H_ values with strong site specificity.

The pristine S-50 edge has the lower H adsorption energy close to neutral. Fe doping keeps the adsorption energies roughly unchanged, while the other dopants lead to worse values. The S-75 edge can be enhanced by Fe and Ni, since the pristine edge adsorbs too strongly. The pristine S-100 edge has adsorption free energy ~−0.5 eV and we predict no enhancement from doping. Finally, it is interesting that although substitutional Fe, Co and Ni at the Mo-50, Mo-100 and S-100 edges all retain their six-fold coordination with respect to surrounding sulfurs, it is hard to find similarities between them in the Δ*G*_H_ values. Therefore, our results corroborate that it is challenging to predict the behavior of adsorption energies based only on the coordination structure of the dopant and the adsorbing sulfur. We observe, curiously, that the behavior of Δ*G*_H_ for Fe, Co and Ni is not smooth as a function of distance from the dopant atom (especially Mo-50 and Mo-100 in Fig. 4). This behavior can be related to the complex, possibly long-range relaxation with the interplay of the atomic and electronic degrees of freedom.

The charged undoped and Fe-doped S-100 edges show some variation in the H adsorption characteristics compared to the neutral charge state (see Table 4). For the undoped case, since ${\rm{\Delta }}{G}_{{\rm{H}}}\approx 0$ eV at the (−1) state, it is in principle possible that at small cathodic overpotentials there are beneficial charge state dependent effects for HER. In contrast, for the Fe-doped edge, none of the adsorption energies at sites 1.−3. is significantly affected in the studied charge states.

**Table 4:** Δ*G*_H_ values for undoped and Fe-doped S-100 edge in charge states +1,…,−2. Results for charge states that are energetically relevant in the range of electron chemical potential 0 eV ≤ *μ*_*e*_ < 0.6 eV are shown.

charge state	undoped S-100	Fe-doped S-100		
		1. nn site	2. nn site	3. nn site
+1	−0.29	—	—	—
0	−0.43	−0.64	−0.35	−0.39
−1	−0.04	−0.66	−0.35	−0.45
−2	−0.53	−0.59	−0.41	−0.43

At the actual HER operating conditions the steady-state hydrogen coverage depends on the exact reaction mechanisms and rates. In a detailed analysis Δ*G*_H_ should thus be evaluated at a system-specific coverage of the surface^11,13,25^. Differential adsorption free energies ${\rm{\Delta }}{G}_{{\rm{H}}}^{{\rm{d}}{\rm{i}}{\rm{f}}{\rm{f}}}$ have been considered in the literature to take this aspect into account^4^. DFT calculations were used to estimate the relevant H coverages for the pristine^25^ and TM-doped edges^7^ in MoS_2_. These calculations suggested both low and high coverages depending on the system, but specifically for Fe, Co, Ni and Cu-doped Mo-50 and for Fe and Co-doped S-100, a low H coverage was reported. Importantly, in the electrochemical characterization of the TM doped edge-terminated nanofilms in Ref.^7^ the Tafel slopes were found in the range (103–118) mV/decade, which is an experimental suggestion that the rate-limiting step in HER is the Volmer step. If this step determined completely the reaction rate, the relevant hydrogen coverage would be close to zero^28^, and our low H-coverage values would thus be the most representative for theoretical interpretation.

To gauge the effect of a larger H coverage we perform some additional tests for absolute and differential adsorption energies (Δ*G*_H_, ${\rm{\Delta }}{G}_{{\rm{H}}}^{{\rm{d}}{\rm{i}}{\rm{f}}{\rm{f}}}$) for pristine Mo-50 at 0.5 monolayer H coverage and for pristine S-100 at 1 monolayer coverage. These edges and coverages correspond to the systems studied by Wang *et al*. Our values for ${\rm{\Delta }}{G}_{{\rm{H}}}^{{\rm{d}}{\rm{i}}{\rm{f}}{\rm{f}}}$ are found in the range (0.0–0.2) eV and (0.3–0.5) eV for Mo-50 and S-100, respectively. In other words, pristine Mo-50 remains close to optimal adsorption conditions even at higher H coverages, and for S-100 the differential adsorption energy is found slightly positive due to stronger hydrogen-hydrogen repulsion effects. Wang *et al*. report the corresponding ${\rm{\Delta }}{G}_{{\rm{H}}}^{{\rm{d}}{\rm{i}}{\rm{f}}{\rm{f}}}$ values 0.06 eV and −0.45 eV, with some discrepancy in the latter value to our results. The discrepancy may be due to the different choice of the supercell, which in our case is a periodically repeating system of vertically oriented layers. Šarić *et al*.^19^ reported recently ${\rm{\Delta }}{G}_{{\rm{H}}}^{{\rm{d}}{\rm{i}}{\rm{f}}{\rm{f}}}$ values for Mo-50 in the case of nanocluster edges and find close to optimal condition at 0.5 monolayer coverage, in agreement with our finding.

In this framework the main question to answer is which kind of doping would improve hydrogen adsorption optimally toward efficient HER on edge-containing MoS_2_ nanostructures. This question is answered in section Discussion.

### Classification and regression analysis

For additional insight machine learning ensemble models (Random Forest, RF) were constructed for the classification and regression tasks for the full dataset containing both basal plane and edge results (see details in Methods) with Δ*G*_H_ as the target quantity. For the classification task, we tested both a tighter and a looser range, ±0.3 eV and ±0.5 eV, respectively. The aim is to obtain a model which predicts whether a given surface structure would lead to a Δ*G*_H_ value for H adsorption in the optimal window, while in regression the model predicts the numerical value of Δ*G*_H_. The objective is to extract more information from the present systematically screened data and assess if a simple approach can be used for future predictions. In addition, the RF models give insight into the importance of the chosen features in explaining factors that affect Δ*G*_H_. Table 5 reports the final RF models’ results. As explanatory features, we include (i) the type of the system (*Type*: basal plane, Mo-0,…), (ii) number of electrons in the outermost valence shell (*Nval*), (iii) coordination number of the dopant (*Coord*, how many sulfurs surround the dopant atom before structural relaxation), and (iv) nearest-neighbor position of the adsorbing sulfur with respect to the dopant atom (*Nn*). The training/test set split is 112/14 cases (see Supplementary Information).

**Table 5:** Results for the RF classification model for two Δ*G*_H_ windows and for the RF regression model. In classification, the accuracy is the percentage of correct predictions of the cases inside/outside of the window. Training, cross-validation and test scores are reported. For cross validation the standard deviation is also given (in parentheses). In regression, the training and test accuracies as *R* ^2^ values are reported. The importance of features is given as output from the final model (see Methods for more details).

Window	Classification		Regression
	±0.3 eV	±0.5 eV	
Accuracy			
*Train*	0.96	0.97	0.90
*Cross-val*.	0.76 (0.04)	0.77 (0.03)	—
*Test*	0.43	0.79	0.42
Importance			
*Type*	0.31	0.29	0.40
*Nval*	0.33	0.29	0.24
*Nn*	0.28	0.34	0.28
*Coord*	0.08	0.08	0.09

The validation accuracy in classification is found to be about 78% for both the broader ±0.5 eV and the narrower ±0.3 eV window. The result for the test set is likely strongly dependent on the samples in the set and the cross-validation score is a better metric for the model’s expected accuracy. For comparison, the cross-validation accuracy for the logistic regression model for the ±0.5 eV window is only about 60%. The level reached by RF in terms of accuracy is very promising since no detailed geometrical features are encoded as input. The importance of features is robust irrespective of the chosen window and the data confirm that the type of the system (basal plane, Mo-0,…) is a significant explaining factor for assessing if the Δ*G*_H_ value is in the chosen window. The number of outermost electrons (*Nval*) and the location of the adsorption site with respect to the dopant (*Nn*) are expectedly important factors. All these three factors are roughly equally important, while the coordination number of the dopant (*Coord*) has little relevance.

**Figure 6:**
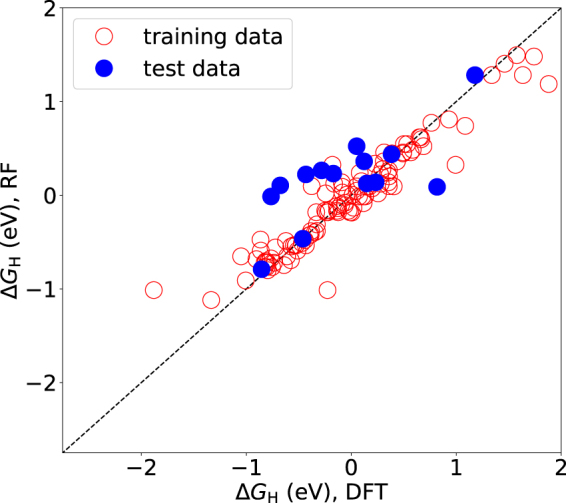
Performance of the RF regression model for Δ*G*_H_.

The performance of the regression model for the training and the test data is shown in Fig. 6. For predicting exact numerical values the model behaves moderately with 0.34 eV mean average error on test data. In the future, the models can be developed further by acquiring more data, encoding the true geometrical structure into features, and studying other algorithms for the learning task. The data set can be also extended to larger H concentrations to predict H coverage effects on Δ*G*_H_.

## Discussion

The results of this work including the output from the machine learning model confirm that the edge type is the most important factor that predicts if the doped system exhibits values in a range around ${\rm{\Delta }}{G}_{{\rm{H}}}=0$ eV at low H coverages. An equally important factor is the type of the dopant. Looking from a general perspective, our findings thus corroborate the importance of two of the proposed avenues of developing better MoS_2_ catalysts^8^: nanostructuring (tailoring the material at the atomic scale toward the most suitable edge structures) and enhancing the internal activity (engineering the structure chemically by doping).

For the 2 H basal plane doping by Fe, Co, Ni and Cu, as well as by Pd and Pt, is predicted to improve without exception HER activity since all of them create favorable local sites for H adsorption in the range −0.35 eV $< \,{\rm{\Delta }}{G}_{{\rm{H}}} < \,0.25$ eV (see Table 1 and summary in Table 6). The formation energy analysis of the charged systems showed that non-neutral charge states are not very likely for the Fe−, Co− and Ni-doped basal plane at electron chemical potentials of interest. Moreover, the supercells in charge state (−1) have Δ*G*_H_ values similar to the neutral ones. The experimental findings by Deng *et al*.^14^ compare interestingly to our predictions. In their experiments the content of Pt, Co and Ni was constant, 1.7 wt% in the MoS_2_ samples, and for Pt they found that single atoms were uniformly dispersed in the plane. They found the Pt-doped basal plane experimentally the most active (our value Δ*G*_H_ = −0.24 eV, see Table 1), Co-doped the second (−0.09 eV) and Ni-doped the last (−0.33 eV). Our Δ*G*_H_ results are clearly in the correct window around 0 eV, but on a detailed level our prediction for Co being the best does not coincide with their finding. This is an interesting discrepancy since any DFT inaccuracies in the Δ*G*_H_ values are not expected to be so large to explain the behavior. In fact, the above finding is possible evidence that the prefactor in the expression for exchange current *i*_0_ may play a crucial role in determining the position of the maximum *i*_0_ with respect to Δ*G*_H_. The prefactor depends on the ratios of the rate constants and can lead to a shift to more negative or positive values from ${\rm{\Delta }}{G}_{{\rm{H}}}=0$ eV^12^. In the current case the results suggest maximum *i*_0_ to be found for ${\rm{\Delta }}{G}_{{\rm{H}}} < 0$ eV, which corresponds to low H coverages and the Volmer reaction being the rate-determining step^12^. This conclusion naturally assumes that there are no other explaining factors, such as additional structural defects, surface damage or non-uniform distribution of the dopants, that determine the order of the experimental HER efficiencies for Pt−, Co− and Ni-doping.

**Table 6:** Suggested transition metal dopants at each system. The doping level is 2.8% (16.7%) for the basal plane (edges). In the upper set, the exact target Δ*G*_H_ = 0 eV is used as selection criterion, while in the lower set the target is Δ*G*_H_ within the given energetic range. In the upper set ‘possible dopants’ are less optimal but lead to better Δ*G*_H_ values than the pristine system. ‘System A/B’ indicates the edges that are reported to coexist in synthesized nanostructure samples in the literature (see text).

	Optimal dopant(s)	Possible dopant(s)	System
Criterion Δ*G*_H_ close to 0 eV			
Basal plane	Co, Cu	Fe, Ni	—
Mo-0	Co	Fe, Ni	—
Mo-50	pristine	—	B/Wang *et al*.^7^
Mo-100	pristine	—	A/Kibsgaard *et al*.^6^
S-50	pristine	—	A/Kibsgaard *et al*.
S-75	Fe, Ni	Co, Cu	—
S-100	—	—	B/Wang *et al*.
Criterion −0.5 eV < Δ*G*_H_ < 0.5 eV			
Basal plane	Fe, Co, Ni, Cu	—	—
Mo-0	Fe, Co, Ni	—	—
Mo-50	pristine, Co, Ni, Cu	—	B/Wang *et al*.
Mo-100	pristine, Fe, Co, Cu	—	A/Kibsgaard *et al*.
S-50	pristine, Fe, Co, Ni, Cu	—	A/Kibsgaard *et al*.
S-75	Fe, Co, Ni, Cu	—	—
S-100	Fe, Co, Ni, Cu	—	B/Wang *et al*.

For the basal plane systematic experimental attempts of doping should thus be continued to understand precisely both the structure-property and the theory-experiment relationship with respect to improving HER efficiency: (i) Exact order of HER efficiencies with respect to theoretical Δ*G*_H_ and the possible shifts of the maximum from the exact ${\rm{\Delta }}{G}_{{\rm{H}}}=0$ eV criterion and (ii) How many active sites each dopant creates. An interesting scenario would be to continue the experimental work by Deng *et al*.^14^ by comparing at least Co, Cu and Fe as dopants and carrying out doping at more than one concentration. The results of such an experiment would considerably help to elaborate the theoretical picture. It can be noted that in experiments some adverse additional effects may come into play: in a recent study it was interpreted that the basal plane becomes covered by Ni atoms or aggregates, and this was likely masking signals of dopant-enhanced electrochemical activity^20^.

For the Mo- and S-edges two sets of results are summarized in Table 6 based on calculations in the neutral charge state of the supercell: (i) Dopants that bring the Δ*G*_H_ values of the pristine edge closer to 0 eV; (ii) Dopants that create adsorption sites with energies in the range −0.5 eV $< \,{\rm{\Delta }}{G}_{{\rm{H}}}\, < \,0.5$ eV. Also the two relevant experimental systems are given: System A (with Mo-100 and S-50 edges) corresponds to nanoclusters by Kibsgaard *et al*.^6^ prepared by physical vapor deposition, and System B (with Mo-50 and S-100 edges) to vertically aligned nanofilms as discussed by Wang *et al*.^7^. For system A’s activity, Kibsgaard *et al*. found Ni-doping to be the best followed by Co-doping, wheres Fe-doped, Cu-doped and pristine nanoclusters exhibited the lowest activity. According to them, doping takes place only at the S-50 edge. For System B’s activity, Wang *et al*. concluded by DFT calculations that the S-100 edge can be activated by doping.

We consider first System A with pristine Mo-100 and doped S-50 edges of the nanoparticles. As discussed earlier, our predictions agree with the preferential doping of S-50 instead of Mo-100. Using the exact criterion Δ*G*_H_ = 0 eV for analysing the increase in HER efficiency upon doping, our data is not in good accordance with the experimental findings. In our calculations both pristine Mo-100 and S-50 already have close to optimal Δ*G*_H_ values, which the doping can possibly only worsen. In fact, recent DFT simulations for the 100% Co-doped Mo-50 edge at the correct finite CoMoS nanoparticle geometry point to optimal differential adsorption energies^19^. Therefore, our computational structures are probably too different from the nanoparticle geometry obtained by Kibsgaard *et al*. for reliable predictions. In a more general perspective on low dopant concentration effects at extended edges, Fig. 4 and Table 6 show that especially Fe, Co and Cu doping (if achievable) at Mo-100, and Fe, Co and Ni doping at S-50 create adsorption sites with energies in the range −0.5 eV $< \,{\rm{\Delta }}{G}_{{\rm{H}}} <$ 0.5 eV.

By turning next to Wang *et al*.’s synthesized MoS_2_ (System B, Mo-50 and S-100 edges), our computational structures resemble closely the actual experimental ones (correct stacking of the layers and similar doping level). For these edges as discussed earlier (Table 3), doping can be expected at both the Mo-50 and S-100 edges. The exact criterion Δ*G*_H_ = 0 eV at our low H coverage case suggests that the activity at Mo-50 cannot be improved, since the pristine edge already satisfies the optimal condition. The exact criterion Δ*G*_H_ = 0 eV cannot thus explain the experimental result that Fe, Co and Ni doping increases HER activity. To reconcile the discrepancy with the experiment, we recall from Fig. 4 that at the Mo-50 edge Fe, Co, Ni and Cu create adsorption sites with −0.7 eV $< \,{\rm{\Delta }}{G}_{{\rm{H}}} < \,0$ eV compared to the 0 eV value at the pristine edge. A similar argument as in the case of the basal plane can now be invoked by considering that the rate constants of the partial reactions of HER may have strongly differing prefactors^12^. In the framework of our results, since experiments clearly indicate doping enhanced HER activity over the pristine system, we must conclude that at the Mo-50 edge there is a possible shift of the maximum position of ${i}_{0}$ toward negative Δ*G*_H_ values. Such a shift corresponds to low H coverages and to Volmer reaction being the rate-determining step, which is indeed suggested by the experimental Tafel slopes on these systems^7^. According to Zeradjanin *et al*.^12^ a negative shift would reduce the achievable ${i}_{0}$ values.

Despite a large body of research on MoS_2_, there is still a vast structural space to screen for optimal configurations for HER and their detailed reaction parameters. In particular, the search space expands when one includes different dopants and doping levels, various hydrogen coverages and any new type of nanostructure (e.g. finite clusters and terraced surfaces, low-dimensional systems). A comprehensive *ab initio* modeling would shed light on the reaction mechanisms of HER. However, these are major tasks especially considering the perspective of materials screening and one may need to resort to more approximative analyses. In addition to Δ*G*_H_, many factors that affect the activity need to be considered in the detailed analysis, such as other atomistic descriptors, effects of water-catalyst interface, substrate, charging and oxidation states of the dopant atoms. Charge state analysis of the undoped S-100 edge showed that cathodic overpotential may induce changes in the system by favoring at some values of electron chemical potential charge states for which H adsorption energy is closer to 0 eV than in the neutral state (see Table 4). However, for the Fe-doped S-100 edge a similar behavior was not found, which suggests that the charge effects are subtle and strongly case specific. It should be thus noted that the detailed charge state and spin multiplicity effects related to doped structures on H adsorption may not be appropriately represented by neutral charge state calculations. The question merits further studies especially in the case of MoS_2_ edges to further refine the oxidation states and the relevant Δ*G*_H_ values. Interesting questions remain also regarding the possible competing H adsorbing sites^29^ (e.g. one deep and one shallow) that can have detailed influence on the HER behavior. Such a more complete analysis of multiple local adsorption sites is beyond the scope of the present work, but have been discussed in our work by Kronberg *et al*.^17^ on site-selective adsorption.

## Conclusions

We have performed a comprehensive analysis of transition metal doping at MoS_2_ surfaces in terms of structures, energetics and hydrogen adsorption characteristics in view of understanding the factors that affect the hydrogen evolution reaction. We study the basal plane and the differently sulfur-terminated molybdenum (Mo-0, Mo-50, Mo-100) and sulfur (S-50, S-75, S-100) edges of the 2H-MoS_2_ polytype. Fe, Co, Ni and Cu are considered as dopants at substitutional Mo sites. We use the Gibbs free energy Δ*G*_H_ of hydrogen adsorption to screen possible HER efficiency improvements upon doping and discuss the results with respect to experimental findings in the literature. For the edge structures, we study doping level of 16.7% and H coverage of 16.7% monolayers (single H adsorption on the edge segment of the supercell). We clarify the relative substitutional energies (RSEs) of the dopant atoms at different edges and find a large variation in the doping affinities. The edges are much easier to dope than the basal plane and the large variation suggests important implications about which edges will become effectively doped in synthesized MoS_2_ nanostructures. Structurewise, Fe, Co and Ni cause only minor or small local restructuring both at the basal plane and at the edges, whereas in some cases Cu leads to stronger deformations, which is likely connected to the occupancy of the localized *d* states.

At the basal plane, hydrogen adsorption energy on sulfur next to the Fe, Co, Ni and Cu dopant atoms is clearly lowered toward optimal adsorption condition (Δ*G*_H_ = 0 eV). Charging of the doped basal plane is found to be neither likely nor influence essentially the Δ*G*_H_ values. At the Mo- and S-edges, doping affects the adsorption energies at the whole edge in a non-trivial way, leading either to beneficial or adverse overall effects. We discuss our findings in detail with respect to experimental cases and identify the potential and challenges in using precise Δ*G*_H_ values to interpret experimental improvements in efficiency. Charging of the edges is an additional degree of freedom that merits further studies. Charging may potentially modify the here reported neutral state H adsorption energies and thereby the HER activity as a function of cathodic overpotential. The present results illustrate that the HER efficiency depends critically and in a subtle way on the edge and dopant distribution in the synthesized nanostructures. We also investigate a machine learning model for predicting Δ*G*_H_ values from a minimal input of the system to bypass the computationally demanding DFT calculation. Already at the minimal level of modeling with a small dataset, the results show a promising accuracy for finding Δ*G*_H_ within a ±0.5 eV window.

The approaches of the present work to predict efficiency improvements in HER would be interesting to apply in the future to other types of materials such as MoC, MoSe_2_ and phosphides. In general, we anticipate that having a large collection of DFT data on hydrogen adsorption characteristics available and emerging automatized predictive algorithms at hand, the design and synthesis of platinum group free electrocatalytic materials will substantially speed up.

## Methods

### DFT calculations

The PBE functional^30^ was used in the density functional theory calculations including the spin polarization. All calculations were performed with the CP2K/Quickstep software^31,32^. Van der Waals interactions were taken into account with the D3 method of Grimme *et al*. with Becke-Johnson damping (DFT-D3(BJ))^33,34^. Double-zeta plus polarization quality molecularly optimized basis sets (MOLOPT-SR-DZVP)^35^ and norm-conserving Goedecker-Teter-Hutter (GTH) pseudopotentials^36–38^ were used. The kinetic energy cutoff was 550 Ry and the cutoff of the reference grid 60 Ry. The Poisson equation for the electrostatic potential was solved assuming periodic boundary conditions. For modeling the basal plane the slab consisted of two horizontal layers with 6 × 6 MoS_2_ units in both the layers. The rectangular cell parameters were (*l*_*x*_ = 15.70, *l*_*y*_ = 21.75, *l*_*z*_ = 26.64) Å. For the Mo- and S-edges the slab model consisted of four vertically oriented layers with 6 × 3 MoS_2_ units in each layer and the cell parameters were (*l*_*x*_ = 18.84, *l*_*y*_ = 24.24, *l*_*z*_ = 27.15) (see Supplementary Information for an example). These lattice parameters correspond to pristine systems and were optimized as described in Ref.^17^ In both the cases the structure repeated periodically in the *x* and *y* directions. A layer of about 9 Å vacuum was used in the non-repeating *z* direction at both sides of the slab. In the calculations of doped systems with or without hydrogens the lattice parameters were fixed to the above values and the atomic positions were optimized using the Broyden-Fletcher-Goldfarb-Shanno algorithm until the force on any atom was less than 0.023 eV/Å.

We calculated the Gibbs free energy of adsorbed hydrogen Δ*G*_H_ as

1 $${\rm{\Delta }}{G}_{{\rm{H}}}={\rm{\Delta }}{E}_{{\rm{H}}}+{\rm{\Delta }}{E}_{{\rm{ZPE}}}-T{\rm{\Delta }}{S}_{{\rm{H}}}\approx {\rm{\Delta }}{E}_{{\rm{H}}}+\mathrm{0.29\ }{\rm{eV}},$$

in which for ${\rm{\Delta }}{E}_{{\rm{ZPE}}}-T{\rm{\Delta }}{S}_{{\rm{H}}}$, the zero-point energy minus the entropic terms, we used the numerical value of 0.29 eV as estimated in ref.^4^ Δ*E*_H_ corresponds to the energy difference

2 $${\rm{\Delta }}{E}_{{\rm{H}}}=\frac{1}{n}[{E}_{{{\rm{MoS}}}_{2}+n{\rm{H}}}-({E}_{{{\rm{MoS}}}_{2}}+\frac{n}{2}{E}_{{{\rm{H}}}_{2}})],$$

where *n* is the number of hydrogen atoms, ${E}_{{{\rm{MoS}}}_{2}+n{\rm{H}}}$ the total energy of the system in which *n* hydrogens have adsorbed, ${E}_{{{\rm{MoS}}}_{2}}$ the total energy of the system before H adsorption, and ${E}_{{{\rm{H}}}_{2}}$ the total energy of molecular hydrogen in the gas phase.

We used the standard formation energy analysis to assess the relative stabilities of doped structures as a function of the charge state^39,40^. Formation energy of a doped system in charge state *q* is given by

3 $${E}_{{\rm{F}}}={E}^{q}+q({\mu }_{e}+{E}_{v})-\sum _{i}{n}_{i}{\mu }_{i}$$

where *E* ^*q*^ is the total energy of the system in charge state *q*, *E*_*v*_ the energy of the valence band maximum, *μ*_*e*_ the electron chemical potential relative to *E*_*v*_, *n*_*i*_ the number of atoms of type *i* and *μ*_*i*_ the the corresponding chemical potential. For *E*_*v*_ we used values calculated with neutral supercells.

### Classification and regression model

We trained a machine learning (ML) ensemble model Random Forests^41^ (RF) to solve two tasks related to predicting the HER activity of a given system: (i) Classification task, which aims to predict if for a given structural input the resulting value of Gibbs free energy of hydrogen adsorption, Δ*G*_H_, is within a chosen window. (ii) Regression task, which aims to predict the actual value of the target variable Δ*G*_H_. The problem of predicting Δ*G*_H_ can be expected to be non-linear (i.e., not necessarily any straightforward connection between structure and Δ*G*_H_ due to many factors, such as long-range rearrangement of the electronic structure induced by the dopant and the hydrogen). For this ML problem, RF model provides a robust method that is considered to avoid overfitting and has been successfully used in cheminformatics (see Ref.^42^ and references therein).

The setup for training the RF model and the selection of features is minimal for the purposes of this work. We work only with very basic features of the studied systems, which do not require DFT or MD simulations. Five features were initially considered: (i) The seven different possible types of the system (*Type*, i.e., basal, Mo-0, Mo-50,… as a categorical variable), (ii) atomic number (*Z*) of the dopant, (iii) number of electrons in the outermost valence shell (*Nval*) of the dopant, (iv) ideal coordination number of the dopant (*Coord*, i.e., how many sulfurs surround the dopant atom before relaxation) and (v) nearest-neighbor position of the adsorbing sulfur site with respect to the position of the dopant (*Nn*). These features can be all considered to affect Δ*G*_H_ to various degree. However, since the features *Z* and *Nval* are strongly correlated, we found that a better accuracy could be obtained, as expected, by dropping *Z* altogether in the model.

The full dataset contains 126 cases in which hydrogen adsorbs on the basal plane or the edge of a doped or the pristine MoS_2_ (see Supplementary Information). The cases were randomly divided into a training set (112 cases) and a test set (14 cases). The RF classification model was trained and the parameters tested with the former set using internal cross-validation with five folds (repeated with different partitions). In testing the parameters the $-\mathrm{0.5\ }{\rm{eV}}\le \,{\rm{\Delta }}{G}_{{\rm{H}}}\le \,\mathrm{0.5\ }{\rm{eV}}$ window was used. We employ Python’s RF estimators (classifier and regressor) as implemented in scikit-learn^43^. In the final RF model two hundred trees were grown and for the other parameters the default values were used. The regression model and the classifier for the narrower window was trained with the same parameters. For the regression model we report the *R* ^2^ value on the training and the test set. The importance of features reported from the RF model is based on the Gini score for the classifier and on the drop in the sum squared error for the regressor.

## Electronic supplementary material

Supplementary Information
