# Supplementary material for: Hydrogen adsorption on doped MoS2 nanostructures
Source: Sci Rep. 2017 Nov 10;7:15243. doi: 10.1038/s41598-017-15622-z (PMC5681643; doi:10.1038/s41598-017-15622-z)
Supplement: Supplementary file 1 — Supplementary Information [file 41598_2017_15622_MOESM1_ESM.pdf]

Hydrogen adsorption on doped MoS_2 nanostructures: Supplementary Information

Mikko Hakala[1,*], Rasmus Kronberg[1], and Kari Laasonen[1]

[1]Department of Chemistry and Materials Science, School of Chemical Engineering, Aalto University, P.O.Box 16100, FI-00076 Aalto, Finland
[*]mikko.2.hakala@aalto.fi

2H basal plane

Additional numerical values are reported for the closest (d_1) and farthest (d_6) nearest-neighbor distance around the transition metal (TM) dopant atoms in doped 2H basal plane of MoS_2 (Table S1) before H adsorption. The distances are also illustrated in Fig. S1 in the case of Co doping. The broken TM...S bond is always found to be that which is in the downward direction (to the sulfur that is on the bottom side of the surface layer). When H adsorbs on a sulfur on the surface, the system becomes H-S...TM, in which the TM...S bond is the broken one.

Table S1. Distance (in Å) from the TM dopant to the closest and farthest S neighbor (d_1 and d_6, respectively).

dopant	d_1	d_6
pristine	2.40	2.40
Fe	2.22	2.31
Co	2.18	3.14
Ni	2.21	3.20
Cu	2.31	3.04
Pd	2.33	3.12
Pt	2.33	3.23

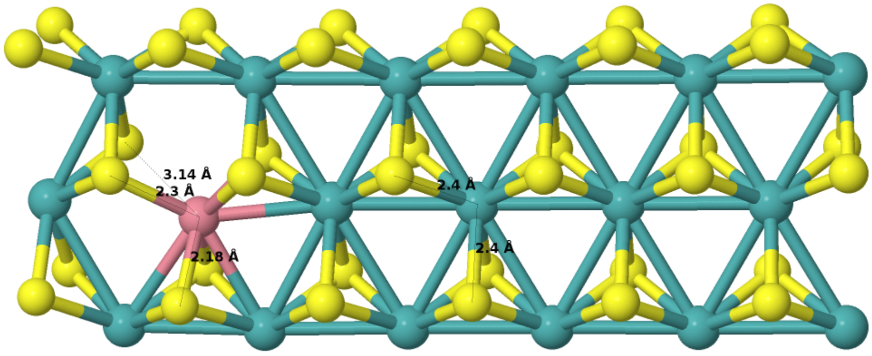

Figure S1. Co-doped basal plane (x-y plane shown, view from the top, along the z axis). The closest and farthest nearest-neighbor distances are indicated for Co as dopant and for Mo as host atom. For Co the shortest nearest-neighbor distance is $d_1 = 2.18$ Å and the longest $d_6 = 3.14$ Å (corresponding to the broken Co...S bond). The nearest-neighbor distances from Mo to its nearest neighbor sulfurs are $d_1 = \cdots = d_6 = 2.40$ Å. As a detail, notice that for cobalt the broken bond Co...S is toward the sulfur at the bottom side of the surface layer.

Excess charge in the simulation cell

The charge state effects on adsorption energies are reported in Table S2 for the basal plane.

Table S2. Effect of excess charge in the simulation cell for ΔG_{H} at the basal plane. The results for the nearest-neighbor adsorption sites are reported (for example Fe...S-H). An excess charge of ± 1 means one electron less or more in the simulation cell of 72 MoS2 units.

dopant	excess charge in cell	ΔG_{H} (eV)
Fe	-2	+0.21
Fe	-1	+0.37
Fe	0	+0.22
Fe	+1	+0.26
Co	-2	-0.01
Co	-1	-0.02
Co	0	-0.09
Co	+1	-0.14
Ni	-2	-0.04
Ni	-1	-0.32
Ni	0	-0.33
Ni	+1	-0.44
Cu	-4	0.10
Cu	-2	-0.07
Cu	-1	-0.30
Cu	0	-0.10

Mo- and S-edges

Effect of doping on local geometry

The changes in the local geometry on doping of the edges is summarized in the main text (Table 2 of the main text). The effects on the structure are discussed here in more detail. To ease the discussion, we divide the six studied edges into three sets from the point of view of nominal coordination number, 4, 5 or 6, of the dopant (coordination number of the substitutional site at the edge). However, this nominal coordination number does not play a relevant role in determining the ΔG_{H} values (see main text).

In the first set, comprising the Mo-50, Mo-100, S-100 edges (see Figure 1 of the main text), Mo is coordinated to six sulfurs similarly to the basal plane. Note, in addition, that in the case of Mo-100 the outermost two sulfurs form dimers. When Fe, Co and Ni are substitutionally doped, we find no essential deformation to the 6-fold coordinated positions. This is in contrast to the basal plane, where Co and Ni doping led to a 5-fold coordinated structure. Quite surprisingly, for Co, Ni, and Fe even the dimerization of the topmost sulfurs in the Mo-100 structure is not affected. In contrast, Cu on Mo-50, Mo-100, S-75 and S-100 edges leads to some deformation of the structure, what is likely connected to its large number of *d* electrons.

In the second set, comprising only the S-75 edge, Mo is coordinated to five sulfurs in the pristine structure and there is a practically a mirror plane which coincides with the MoS_2 sheet. Doping with Co and Ni preserves the coordination and the mirror symmetry, while doping with Fe preserves the coordination but leads to slightly reduced symmetry. Doping with Cu leads to some structural deformation. Finally in the third set, comprising Mo-0 and S-50, Mo is coordinated to four sulfurs. For the Mo-0 edge, Fe, Co Ni and Cu preserve practically fully the structure and coordination compared with the pristine structure. For the S-50 edge, Fe, Co and Cu doping preserve the coordination and the structure, whereas Ni leads to a slight symmetry change.

Example of the computational system

Figure S2 gives an example of the computational system for single hydrogen adsorption on doped Mo-50 edge (for illustration the supercell is extended to 2 x 2 size). In this example hydrogen adsorbs on the second-nearest neighbor sulfur site with respect to the position of the dopant atom.

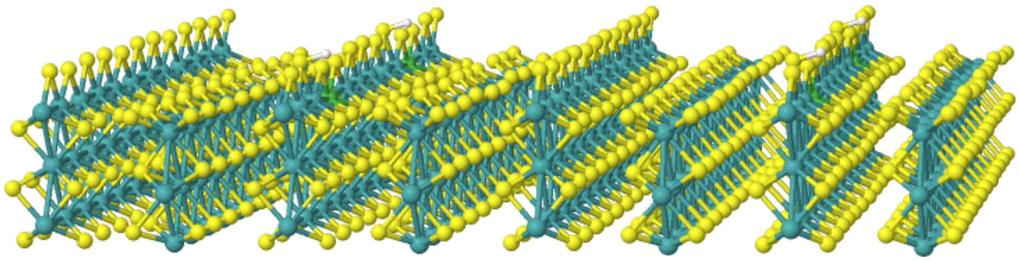

Figure S2. Example of the computational system in the case of H adsorption on Ni doped Mo-50 edge at the second-nearest neighbor sulfur site. The actual supercell is 229 atoms, which is here repeated to 2 x 2 size in the *xy* plane for illustration.

Gibbs free energy of adsorption ΔG_{H}

Details of the dataset

Table S3 shows the distribution of the cases in the dataset as a function of dopant and system type.

Table S3. Details of the dataset. Upper part: Number of cases in the dataset that contain a given dopant. Lower part: Distribution of systems in the dataset.

	number	
dopant		
pristine	10	
Fe	23	
Co	24	
Ni	29	
Cu	21	
Pd	10	
Pt	9	Total: 126
system		
basal plane	15	
Mo-0	14	
Mo-50	13	
Mo-100	24	
S-50	19	
S-75	25	
S-100	16	Total: 126

Dataset

Columns of the dataset:

- 1 / Type of system
- 2 / Dopant ('pr'≡ pristine)
- 3 / Index of nearest-neighbor sulfur to which H adsorbs relative to the site of the dopant
- 4 / ΔG_{H} (eV)

```
basal pr 1 1.880
basal Fe 1 0.224
basal Co 1 -0.085
basal Co 3 1.740
basal Ni 1 -0.334
basal Ni 1 -0.012
basal Ni 1 -0.126
basal Ni 2 1.338
basal Ni 2 1.178
basal Ni 4 1.460
basal Ni 10 1.576
basal Cu 1 -0.097
basal Pd 1 -0.223
basal Pt 1 -0.240
basal Pt 2 1.634
Mo-0 pr 1 -0.862
Mo-0 pr 1 -0.513
Mo-0 Fe 1 0.198
Mo-0 Fe 2 -0.825
Mo-0 Fe 3 -0.855
Mo-0 Co 1 -0.040
Mo-0 Co 2 -0.814
Mo-0 Co 3 -0.797
Mo-0 Ni 1 0.358
Mo-0 Ni 2 -0.904
Mo-0 Ni 3 -1.006
Mo-0 Cu 1 1.085
Mo-0 Cu 2 -0.677
Mo-0 Cu 3 -0.865
Mo-50 pr 1 0.003
Mo-50 Fe 1 -0.623
Mo-50 Fe 2 -0.723
Mo-50 Fe 3 -0.765
Mo-50 Co 1 -0.328
Mo-50 Co 2 0.021
Mo-50 Co 3 -0.328
Mo-50 Ni 1 -0.384
Mo-50 Ni 2 -0.126
Mo-50 Ni 3 -0.442
Mo-50 Cu 1 0.347
Mo-50 Cu 2 0.096
Mo-50 Cu 3 -0.213
Mo-100 pr 1 -0.061
Mo-100 Fe 1 0.132
Mo-100 Fe 2 0.173
```

Mo-100 Fe 3 -0.061
Mo-100 Fe 4 0.012
Mo-100 Co 1 -0.137
Mo-100 Co 2 0.196
Mo-100 Co 3 0.130
Mo-100 Co 4 0.052
Mo-100 Ni 1 -0.737
Mo-100 Ni 2 0.073
Mo-100 Ni 3 -0.755
Mo-100 Ni 4 -0.026
Mo-100 Cu 1 0.152
Mo-100 Cu 2 0.147
Mo-100 Cu 3 0.117
Mo-100 Pd 1 -0.798
Mo-100 Pd 2 -0.765
Mo-100 Pd 3 -0.806
Mo-100 Pd 4 -0.249
Mo-100 Pt 1 -0.814
Mo-100 Pt 2 0.108
Mo-100 Pt 3 -0.824
Mo-100 Pt 4 -0.136
S-50 pr 1 0.050
S-50 pr 1 0.404
S-50 Fe 1 0.080
S-50 Fe 2 0.122
S-50 Fe 3 -0.172
S-50 Co 1 0.493
S-50 Co 2 0.585
S-50 Co 3 0.383
S-50 Ni 1 0.354
S-50 Ni 2 0.565
S-50 Ni 3 0.296
S-50 Cu 1 0.930
S-50 Cu 2 0.763
S-50 Cu 3 0.387
S-50 Pd 1 -0.092
S-50 Pd 2 0.313
S-50 Pt 1 0.232
S-50 Pt 2 0.494
S-50 Pt 3 0.297
S-75 pr 1 -1.050
S-75 pr 1 -0.612
S-75 Fe 1 0.687
S-75 Fe 1 0.051
S-75 Fe 2 -0.283
S-75 Fe 2 0.358
S-75 Fe 3 0.280
S-75 Fe 3 -0.163
S-75 Co 1 0.660
S-75 Co 1 0.648
S-75 Co 2 -0.378
S-75 Co 2 0.357
S-75 Co 3 0.993
S-75 Co 3 -0.180
S-75 Ni 1 0.817

```
S-75 Ni 1 -0.084
S-75 Ni 2 -0.433
S-75 Ni 2 0.275
S-75 Ni 3 0.255
S-75 Ni 3 -0.245
S-75 Cu 1 0.532
S-75 Cu 1 0.501
S-75 Cu 2 0.666
S-75 Cu 3 0.309
S-75 Cu 3 0.435
S-100 pr 1 -0.433
S-100 Fe 1 -0.642
S-100 Fe 2 -0.347
S-100 Fe 3 -0.387
S-100 Co 1 -1.335
S-100 Co 2 -0.471
S-100 Co 3 -0.530
S-100 Ni 1 -1.882
S-100 Ni 2 -0.458
S-100 Ni 3 -0.527
S-100 Cu 1 -0.576
S-100 Cu 2 -0.398
S-100 Cu 3 -0.572
S-100 Pd 1 -0.227
S-100 Pd 2 -0.463
S-100 Pd 3 -0.547
```
